# Evaluating the long-term impact of the Fostering Changes training programme for foster carers in Wales, the Confidence in Care trial: study protocol for a randomised controlled trial

**DOI:** 10.1186/s13063-017-2424-3

**Published:** 2018-01-11

**Authors:** Gwenllian Moody, Lucy Brookes-Howell, Rebecca Cannings-John, Sue Channon, Elinor Coulman, Mandy Lau, Alyson Rees, Jonathan Scourfield, Jeremy Segrott, Michael Robling

**Affiliations:** 1 Centre for Trials Research, Cardiff University, Neuadd Meirionydd, Heath Park, Cardiff, Wales UK; 2 Children’s Social Care Research and Development Centre (CASCADE), School of Social Studies, Cardiff University, Cardiff, Wales UK; 3 Centre for the Development and Evaluation of Complex Public Health Interventions for Public Health Improvement (DECIPHer), Cardiff University, Cardiff, Wales UK

**Keywords:** Foster, Fostering Changes, Training, Foster carer, Randomised controlled trial, Looked-after children, Self-efficacy, Complex intervention

## Abstract

**Background:**

The Fostering Changes programme was developed by the Adoption and Fostering National Team at the Maudsley Hospital, South London, in conjunction with King’s College London. It is a 12-week group-based training programme for foster and kin carers, which aims to build positive relationships between carers and children, encourage positive child behaviour and set appropriate limits, through a practical skills-based approach. The programme also aims to improve foster carers’ understanding of the causes of children’s social and emotional difficulties and their confidence in applying this knowledge in various situations.

**Methods:**

This is a pragmatic open-label individually randomised controlled trial, with embedded process evaluation. A total of 237 participants will be recruited from Welsh Local Authorities and Independent Fostering Providers; those allocated to the intervention group will be offered enrolment in the next Fostering Changes programme group at their site. Participants in the control group will be offered the Fostering Changes programme at the end of the follow-up period. Data will be collected at baseline, immediately following the 12 week Fostering Changes intervention, and 12 months from the start of the Fostering Changes programme. The primary outcome measure assesses the extent to which carers feel able to cope with and make positive changes to the lives of their foster children and is measured by the Carer Efficacy Questionnaire at 12 months.

**Discussion:**

The trial will determine whether the Fostering Changes programme, in the long term, can deliver important, significant differences to the way foster carers build positive relationships with their foster children, encourage positive child behaviour and set appropriate limits, compared with usual care.

**Trial registration:**

International Standard Randomised Controlled Trial Number, ISRCTN19090228. Registered on 11 January 2017.

**Electronic supplementary material:**

The online version of this article (10.1186/s13063-017-2424-3) contains supplementary material, which is available to authorized users.

## Background

### Background and rationale

There were 5660 ‘looked-after’ children and young people in Wales on 31 March 2015 [1], many of whom are being cared for by foster carers or relatives (‘kin carers’). Children in care are children who are ‘looked after’ by a local authority under the Children Act 1989 and the Social Services and Well-being Act 2014. Looked-after children and young people are more likely to have difficulties related to emotional well-being, mental health [2] and education [3, 4] than are other children and young people who are not in care and not in need. This can place a strain on carers and increase the likelihood of placement disruption [5]. The importance of continuity of care for looked-after children has long been established [6, 7].

In Wales, new carers must undertake pre-approval and later induction training and can then take optional further training [8]. Training is normally provided by the local authority or independent fostering providers. Skill-based training may aim to provide carers with support on such topics as developmental needs and techniques to manage difficult emotions and behaviours [9]. There have been a variety of ways in which group-based UK training programmes have sought to support foster carers, including helping carers to deal with common challenges. These may cover a variety of concepts, including attachment, managing challenging behaviour, carer confidence and communication skills. Despite numerous evaluations of such training there have been few attempts at assessment via a formal randomised controlled trial. A trial of group training on communication and attachment by Minnis et al. [10] demonstrates some of the key challenges in this field, with high rates of withdrawal and high rates of programme attrition, high baseline levels of psychopathology amongst looked-after children and no differences found at 9 months follow-up. A second trial [11] with a shorter level of follow-up similarly found few differences attributable to a cognitive behavioural training programme for carers, including training on placement stability.

The Fostering Changes programme was developed by the Adoption and Fostering National Team at the Maudsley Hospital, South London, in conjunction with King’s College London [12]. The programme aims to build positive relationships, encourage positive child behaviour and set appropriate limits, through a practical skills-based approach. Additionally, the Fostering Changes programme aims to improve foster carers’ understanding of the causes of children’s social and emotional difficulties and their confidence in applying this knowledge in various situations [12]. The Fostering Changes programme comprises weekly 3 hour sessions held over 12 weeks, is based on social learning theory [13] and attachment theory [14] and was developed using ideas from other parent-training programmes [15]. Before-and-after evaluations of earlier versions of the training programme found some improvements, including improved carer–child interaction, reduced carer stress and fewer carer-reported child problems [15]. With increased understanding of how neglect and abuse impacts children’s development, the programme was further modified to place more emphasis on attachment relationships and how to support carers in improving the educational outcomes of looked-after children [12].

Briskman et al. [12] trialled the revised programme and found significant improvements in indices of child and young person behaviour, carer-defined problems, emotional and behavioural difficulties and quality of relationship in the intervention group compared with a control group. While encouraging, all outcomes were assessed immediately following programme delivery at 12 weeks; therefore, it is not known whether these potential benefits endure over time. The Briskman et al. [12] trial was conducted with a sample of 63 carers across four Greater London local authorities; it is also important to investigate the effectiveness of the programme in a more representative sample of foster carers from a wider geographic area. While the outcomes of the Briskman et al. [12] trial have been used to justify the on-going roll-out of the programme in both England and Wales, it is important to establish independently replicated findings of programme effectiveness to support broad implementation.

## Methods/design

### Objectives

The objective of the trial is to assess the longer-term effectiveness of the Fostering Changes programme in supporting foster carers. The trial will provide the first evidence of programme effectiveness beyond the end of the training programme.

### Trial design

This study is a pragmatic open-label individually randomised controlled trial, with embedded process evaluation. Participants recruited to the intervention group will be offered enrolment in the next Fostering Changes programme group at their site. Participants in the control group will be offered the Fostering Changes programme at the end of the follow-up period. All participants will continue to have access to usually provided support and advice services.

### Study setting

The Confidence in Care consortium is funded by the Big Lottery Fund to deliver Fostering Changes across all local authorities in Wales. The four Confidence in Care delivery partners will deliver the Fostering Changes programme to each provider agency; either local authorities or independent fostering providers. Provider agencies will have a nominated staff member responsible for the running of the programme in that site. A total of 19 sites will be set up. Site set-up is led by the trial manager and can be conducted either face-to-face or remotely. Set-up involves coordination with local nominated staff, a presentation about trial aims and methods, fulfilling requirements from the site (e.g., provision of pseudonymised lists of eligible carers, distribution of study packs to carers) and resolving queries.

### Site selection

The Fostering Changes programme aims to run in all 22 local authorities in Wales as well as with some independent fostering providers. The roll-out of the programme was determined in a delivery framework, set out at the start of the Fostering Changes programme. While the framework provides an overarching structure, there is some flexibility over which sites are used when. The sites selected to be part of the randomised controlled trial will be all sites used between January 2016 and April 2017 (inclusive). Some trial providers may deliver more than one training course in this period. All sites will be included except those that are deemed to be too small (i.e., insufficient likely eligible participants) to accommodate the randomised controlled trial.

### Participant selection

Participants can either self-select by responding to a postal invite or be nominated by provider agencies. Provider agencies will select participants to nominate based on locally determined criteria, including perceived needs of a foster carer, or apparent availability based on absence of competing commitments.

### Eligibility criteria

Eligibility criteria will match Fostering Changes programme enrolment criteria. Participants in the trial must be local authority foster carers or employed by an independent or not-for-profit agency or be family carers (kin carers and non-related foster carers), currently have a child aged two or over placed with them and expect to be caring for that child for the duration of the Fostering Changes programme (i.e., at least 12 weeks). Foster carers must be prepared to attend all 12 sessions of the programme and they must have sufficient understanding of English or Welsh to complete the intervention.

Participants must not have not attended the Fostering Changes programme previously, not have a foster child attending a children’s skills group (a separate intervention being delivered by the Confidence in Care consortium outside of the trial), and must not live in the same household as another carer who has participated in the Fostering Changes programme.

### Interventions

#### Trial intervention: the Fostering Changes programme

Participants in the intervention arm will receive the 12 week group-based training programme (delivered in line with school terms), and three termly support group sessions designed to reinforce and maintain programme learning. The programme has been commissioned to run with a group size of 12 carers, which may involve a mix of foster and kin carers. Week one of the training programme is an induction session; thereafter, each session will last 3 hours and will consist of:

- Start of session: feedback from carers regarding skills covered during the previous week and experience at home
- Review of theoretical material underlying the topics (see Table 1) for that week (in a way that is accessible for carers)

**Table 1:** Summary of weekly topics [30]

Week	Contents: topics covered
1	Establishing the group
	How children thrive Experiences of looked-after children Developmental stages Tracking and observing behaviour
2	Context of behaviour
	Attachment: child and carer Social learning theory ABC: triggers and pay-offs
3	Praise
	Needs and behaviour Positive strategies Praise Targeting an alternative behaviour
4	Positive attention
	Praise for education Praise to support learning Praise for being or doing Play Attending
5	Communication skills
	Communicating with looked-after children Identifying communication skills Regulating emotions When listening is difficult Reflective listening and questions
6	Context of education
	Context of education Creating a good learning environment Supporting homework Positive strategies
7	Reading friendly
	Expressing feelings Reading friendly Rewards
8	Giving instructions and ignoring
	Good instructions Ignoring Assertiveness
9	Discipline
	Reminiscence Positive discipline Family rules Natural and logical consequences
10	Time out and problem solving
	Attending more than one child Time out Problem solving Stop – plan – go
11	Endings and review
	Capturing your child’s time with you Precious things Facilitating positive endings Fostering flower power Attending recap – more than one child
12	Relaxation and going forward
	Taking care of yourself Stop – plan – go: future strategies What I appreciate about you Certificate giving Final party
- New skills or strategies to be used at home
- End of session feedback

The programme will be delivered by four delivery partners: The Fostering Network, Barnardo’s, Action for Children and The Adolescent and Children’s Trust. Programme delivery is independent of the research team, although a delivery group (delivery partners and research team) will meet to coordinate the Fostering Changes programme roll-out within the trial. Delivery partners will provide data for the process evaluation (e.g., attendance data).

Fostering Changes facilitators are selected by delivery group members based on a best-practice guide issued by the SLAM team. All facilitators can choose to be accredited in the delivery of the programme by the South London and the Maudsley (SLAM) team, but are under no obligation to do this.

### Usual services

There is no active control. Usually provided support and advice services include, but are not restricted to, support from the local fostering team, access to The Fostering Network helpline, universal health and education services and locally organised foster carer support groups.

### Retention strategy; adherence

To maintain engagement, encourage retention and thank foster carers, trial participants will receive a £10 high street voucher on completion of the 3 month and 12 month questionnaires. Contact details will be collected during recruitment and will be verified by provider agencies as the trial continues. Participants will be reminded via text and email when a questionnaire will be posted to them and will also receive a newsletter updating them on study progress at 9 months post-recruitment. Participants who do not respond to postal follow-up will be contacted with the offer to complete their follow-up questionnaires via telephone if they wish. Participants have the right to withdraw consent for participation at any time.

### Outcome measures and participant timeline

#### Primary outcome measure

The primary outcome measure was selected to assess the extent to which carers feel able to cope with and make positive changes to the lives of their foster children and is measured by the Carer Efficacy Questionnaire (CEQ) at 12 months. The primary comparative analysis will employ an analysis of covariance (ANCOVA) model to the CEQ score (adjusting for baseline CEQ score) to investigate intervention effect. The CEQ was designed by the clinical team at King’s College London [12] and contains nine Likert-like items relating to knowledge, ability and possible change, which are assessed using a five-point response scale ranging from strongly disagree to strongly agree. A higher total score indicates stronger beliefs about the carer’s own ability to make positive changes to children’s behaviour and outcomes. This scale was included in the pilot phase that preceded the main Briskman trial [12], which, amongst other things, established acceptability e.g., in terms of matters such as completion time. Content validity was established through the input of the Kings clinical team (i.e., through their specification of the three stated content domains). Briskman et al [12] found a non-significant difference in CEQ scores between the study groups favouring the intervention arm, which, given the small sample size, provides some support for concurrent validity. The internal consistency of this scale, assessed using Cronbach’s alpha, was 0.66, indicating satisfactory internal validity. However, the current researchers would agree that the evidence for scale validity requires further work. We will explore missingness and floor or ceiling effects for the items contributing to the primary outcome measure.

#### Secondary outcome measures

Secondary objectives are listed as follows:

- To ascertain whether the *Fostering Changes* programme makes a difference to rates of unplanned moves for looked-after children
- To ascertain whether the *Fostering Changes* programme makes a difference to looked-after children’s reported engagement with education at 12 month follow-up
- To ascertain whether the *Fostering Changes* programme makes a difference to carer’s support for child’s education at 12 month follow-up
- To ascertain whether the *Fostering Changes* programme improves carer–child relationships at 12 month follow-up
- To ascertain whether the *Fostering Changes* programme improves carers’ coping strategies
- To ascertain whether the *Fostering Changes* programme improves child behaviour and emotional problems
- To ascertain whether the *Fostering Changes* programme reduces carer-defined problems
- To assess the use of services and support
- To conduct a process evaluation, including participant and facilitator interviews and intervention observations, to examine contextual factors, causal mechanisms and fidelity of programme intervention

**Fig. 1:**
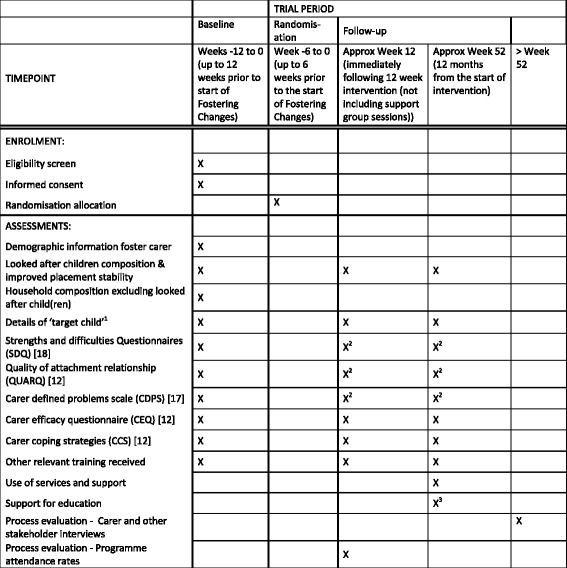
Participant timeline: schedule of enrolment, interventions and assessments (SPIRIT figure, see Additional file 1 for SPIRIT checklist)

More details are given in Fig. 1. Potential moderators will be examined, including socio-demographics and carer and foster child history recorded at baseline.

The Carer-Defined Problems Scale [16] is an individualised measure of carer-nominated child behavioural problems. The Strengths and Difficulties Questionnaire (SDQ) [17] measures a child’s social, emotional and behavioural adjustment. The Carers’ Coping Strategies scale assesses carers’ coping strategies and the Quality of Attachment Relationship Questionnaire is a measure of carer–child attachment relationship. The last two measures were developed by the Briskman et al. [12] trial team.

### Sample size estimation

The sample size was informed by the reported Carer Efficacy Scale results from the previous trial [12]. Based on a mean [18] score of 27.1 (3.9) in the control group, and a 2:1 ratio (intervention: control) to maximise the number of carers attending each training programme, a sample size of 213 carers (142:71) would provide 80% power at the 5% level to detect a difference of 1.6 points on the Carer Efficacy Scale. To allow for 10% loss to follow-up (owing to non-response), the sample size is inflated to 237 (158:79 respectively). Participants will be recruited at an average rate of 14 per month for 17 months (although it is recognised that recruitment will be termly in nature). This sample size is based on a maximum of one carer being recruited per household. This does not prevent two carers from the same family attending the training sessions but only one would be a trial participant. Households will be asked to identify the primary carer for the purposes of evaluation in such circumstances.

### Data collection methods

#### Recruitment and consent

Recruitment will occur in waves aligned to the school terms (three rounds of recruitment are planned per year). Each local authority will supply the trial team with a list of pseudonymised foster carer details who meet study eligibility criteria. For each site or wave, at least 50 carers will be randomly selected to receive a study pack containing an invitation letter, a participant information sheet, local information, including the dates and venue of the Fostering Changes programme, a Fostering Changes leaflet describing the contents of the programme, and a reply slip with a stamped-addressed envelope. Carers can register their interest by responding to the trial team via postal reply slip, email, phone or text. Both local authorities and independent fostering providers will further provide to the trial team a subset of at least 18 eligible foster carers considered to be both interested and suitable to take part in the trial, who have provided consent to be contacted by the trial team.

Foster carers will be contacted by Health and Care Research Wales researchers or a member of the core trial team to explain the trial, answer any questions and confirm eligibility. Carers will then be offered either a telephone interview or a home visit and told about further details of the trial and the Fostering Changes programme, and given an opportunity to address any questions they may have. If they agree to participate in the trial, written consent (at the home visit) or oral consent (during the telephone interview) will be obtained and baseline measures taken. For those who complete a telephone interview, written consent will also be sought at a later date if possible.

Once sufficient carers have been recruited to allow formation of one group (*n* = 18), recruitment will stop for that site or wave. The trial team will inform the provider agency and the delivery team of who has been recruited.

#### Frequency and duration of follow-up

Data will be collected by Health and Care Research Wales researchers or trial team researchers at baseline, and by trial team researchers at the end of the 12 week Fostering Changes programme (termed 3 month follow-up) and again 9 months following this (termed 12 month follow-up). Baseline data will be collected at home visits or by telephone interview up to 3 months before the start of the Fostering Changes programme. Follow-up data will be collected via postal questionnaires or by telephone interview. In the first instance, postal questionnaires will be sent out to participants; if there is no response in 2 weeks, members of the trial team will attempt to complete a telephone interview with the participant.

To reduce the risk of bias in data collection, trained data collection staff will use a ‘script’ when contacting participants for follow-up. Participants will be asked not to reveal their allocation to data collection staff until the end of interview, when a question about programme attendance is asked. If allocation is revealed to data collection staff before this point, this will be noted. The formal follow-up strategy will include such details as how participants are to be contacted, how many times, ways to increase follow-up rates through the use of newsletters to ensure that participants are engaged with the trial, and the updating of participant contact details from provider agencies. All data will be captured on paper case report forms and will be entered manually in a database, hosted on secured university servers. A 10% check of the data will be made to ensure that data have been entered correctly. Programme attendance data will be collected by programme facilitators and supplied to the research team via the Confidence in Care delivery team.

#### Randomisation; sequence generation

Carers agreeing to participate will be allocated to a trial arm with an assignment ratio 2:1 (intervention to control group) using an algorithm that will minimise by type of carer (kin carer or unrelated carer) and age of looked-after children in the household (<12 or ≥ 12 years) for each site.

Following initial site set-up, participants may be recruited at any time-point before the first Fostering Changes group is due to commence. Allocation will only occur within 6 weeks of the start of the Fostering Changes programme and will be conducted centrally by the core trial team via telephone following notification of recruitment. There will be complete concealment of the preceding allocations from the field recruitment staff, eliminating the possibility of predicting the next allocation. Participants will be telephoned by the trial manager and informed of their allocation. With the exception of the trial manager and study administrator, all trial team members and field recruitment staff will be blinded to allocation. The trial statisticians will be unblinded after introduction of the allocation by nature of the 2:1 allocation ratio. Control-allocated carers will be able to access training after end of study period.

### Data management

Data will be entered on paper case report forms at site, staff will be trained in good clinical practice and study-specific procedures. Clinical data will then be entered manually into a SQL database by the trial administrator. Study management data will be entered into an MS Access database by the trial administrator. Both clinical and study management data will be checked visually on receipt by the trial administrator, automatic validation checks will be completed during clinical data entry (built into the SQL database) and 10% of all data manually entered into the SQL database will be checked by the data manager; finally, data will be checked during data cleaning using SPSS Syntax for validations and missing data. Only the core trial team members will have access to hard copies of the data, and these will be stored in a locked cupboard. The same individuals will have access to the study management database and the SQL database, which will both be password-protected. A data management plan will be completed and adhered to.

### Statistical methods

#### Main analysis

This protocol paper follows SPIRIT guidelines, and the analysis and reporting of this pragmatic randomised trial will be in accordance with CONSORT guidelines. Data will be analysed using the intention-to-treat principle; all eligible randomised participants will be included in the analysis. The primary comparative analyses will employ an ANCOVA model to the CEQ score (adjusting for baseline CEQ score) to investigate intervention effect at 12 months. Additional covariates included are those balanced at randomisation (type of carer and age of looked-after children in household). Although ANCOVA is robust against normality violation [19, 20], homoscedasticity will be explored using various diagnostic plots of the residuals of the fitted model (such as kernel density estimators and quantiles of the residuals against quantiles of normal distribution). If there is evidence of heteroscedasticity amongst the residuals then robust standard errors will be used. Mixed-effects three-level regression models will be used to adjust for site (local authority or independent fostering providers) as a stratification variable and to allow for clustering by block in the intervention group. The result will be presented as the (adjusted) difference in mean CEQ score between the intervention and control groups, along with 95% confidence interval and *p* value, with due emphasis placed on confidence intervals for the between-arm comparisons. A secondary analysis of the primary outcome will examine CEQ score over time incorporating the 3 month response.

Secondary outcome measures will be analysed using multilevel linear or logistic regression at 3 and 12 months follow-up. Baseline reported scores, and variables balanced at randomisation will be controlled for as covariates. Various hierarchies will be investigated to explore clustering. Repeated measures models will include an interaction term for time and trial arm to investigate any divergent or convergent pattern in outcome measures [21]. The adjusted regression coefficients will be provided alongside 95% confidence intervals and *p* values. The categorical version of SDQ total difficulties will be compared between trial arms by fitting a multilevel ordinal regression model to the SDQ category at 3 and 12 months follow-up. Baseline reported SDQ score, and variables balanced on at randomisation will be controlled for as covariates.

With validated scales, the outcome will be used as directed in the manuals, using either categorical (using validated cut-offs) or continuous score. Newly derived or modified measures will be further validated using Cronbach’s α to assess the scale reliability; a factor analysis will determine factor loadings.

#### Subgroup and interim analysis

There is no planned interim analysis. Exploratory subgroup analyses are planned for:

- Age, sex, placement history of the index foster child
- Experience, qualifications of the foster carer and size of household

Appropriate interaction terms will be entered into the primary regression analysis to conduct pre-specified subgroup analyses. Subgroups will be fully defined in advance of any analysis being started with input from the trial team and summaries of current evidence from the literature. Since the trial is powered to detect overall differences between the groups rather than interactions of this kind, the results of these exploratory analyses will be presented using confidence intervals. A full statistical analysis plan will be completed and signed off prior to any analysis being undertaken.

### Data monitoring

Regular monitoring will be performed by the trial or data manager according to the principles of good clinical practice. Data will be evaluated for compliance with the protocol and accuracy. Following written standard operating procedures, the monitors will verify that the trial is conducted and data are generated, documented and reported in compliance with the protocol. There is no separate data monitoring committee for the trial. Any key data queries will be taken to the trial steering committee.

### Adverse event monitoring

There are no expected adverse events related to the intervention or research procedures, and the Cardiff University School of Social Sciences (SOCSI) Research Ethics Committee have approved that they should not be reported for this trial.

### Auditing

No independent audits are planned.

### Ethical and governance approval

Ethical approval for this trial was given by Cardiff University SOCSI Research Ethics Committee on 4 June 2015, reference number SREC/1515; this was centralised ethical approval for all sites. A trial steering committee will meet approximately every 6 months to provide study oversight. The trial steering committee comprises two independent academic social workers (one of whom is the chair), an independent statistician and a lay representative. The Fostering Changes programme was selected for use in the Confidence in Care project following input from a multi-sector advisory group, which included representation from heads of children’s services in Wales.

### Confidentiality

All data will be kept for 15 years, in line with Cardiff University’s Research Governance Framework Regulations for clinical research. Electronic data will be stored confidentially on password-protected servers maintained on the Cardiff University Network. All hard copy forms will be stored in locked filing cabinets. For participant interviews, all audio files will be recorded on encrypted audio-recorders and securely held in password-controlled databases on Cardiff University servers. Audio files will be transcribed and anonymised using university-approved transcription companies. No identifiable data will be published.

### Access to data

The chief investigator will have access to the final trial dataset.

### Dissemination policy

A publication plan and dissemination policy will be written. The trial results will be disseminated in full and with a lay summary on the Centre for Trials Research website, and a summary of the results will be disseminated to all participants. It is expected that all study management team members (protocol paper co-authors) will co-author the main results paper. Any data requests should be made to the Centre for Trials Research, which is a signatory of AllTrials and aims to make its research data available wherever possible.

### Process evaluation

An integral process evaluation of this complex training intervention will aid interpretation of the trial results. The process evaluation will mainly be undertaken through qualitative interviews with foster carers, training facilitators and other stakeholders. We will aim to triangulate the qualitative data on experiences of the intervention with quantitative data on attendance throughout the 12 week intervention. This is likely to include exploration of programme enrolment, quality of engagement, retention and reach.

The process evaluation will take part in two phases. The first phase will involve further development of the logic model of the intervention. The theoretical underpinnings of the training have already been proposed and, along with the initial Briskman et al. [12] trial, there is some evidence for how theoretical change mechanisms may lead to effect (hence, the selection of carer efficacy as primary trial outcome measure). Nevertheless, an explicit logical model has yet to be developed. A detailed understanding of the mechanisms of the intervention is necessary for us to explore whether the Fostering Changes programme was delivered as originally intended. The logic model will be developed through review of programme materials and refined through interaction with intervention designers. In addition, it is expected that there may be some variation in support routinely provided to carers throughout Wales. To better understand the nature of the comparison being made in the trial and how the study setting may influence outcomes, the first phase will also include an assessment of usual support for foster carers. We will use a mixed-methods approach to combine documentary analysis of key existing documents, qualitative data from interviews with foster carers and quantitative questionnaire data with foster carers about other support accessed.

Using qualitative interviews with foster carers (*n* ≤ 40), interviews with Fostering Changes facilitators and training providers (*n* ≤ 15), and quantitative measurements (including attendance rates), the second phase of the process evaluation (Table 2) will explore:

**Table 2:** Summary of key process evaluation components (phase 2)

Component	Set-up work	Within-trial baseline, outcome assessment	Routine data collection (group training)	Exit interviews (after 12 month follow-up): carers or training providers
Fidelity	Y		Y	Y
Contamination			Y	Y
Assessing usual care	Y	Y	Y	Y
Acceptability				Y
Feasibility				Y

1. Fidelity of the intervention: We will develop intervention fidelity measures and assess whether the Fostering Changes programme was delivered as originally intended (e.g., quantify session attendance, qualitatively explore facilitator approach to delivery of manualised programme content) [22] and, therefore, determine whether the trial represents a good test of the intervention. We will explore barriers to, and facilitators of, optimal implementation.
2. Acceptability of the intervention: We will explore the acceptability of the training intervention to carers; in particular, how the new elements of the programme (follow-up carer support) were experienced by carers.
3. Feasibility of intervention implementation: This will include assessing factors that may impact implementation of Fostering Changes through interviews with training providers and other key stakeholders (e.g., leads from local authority fostering teams).
4. Contamination within trial: We will monitor using group session attendance records and participant follow-up questionnaires and through process evaluation interview exposure of control group participants to the intervention.

Interview schedules (and where relevant, topic guides) will be developed by the research team informed by the research aims and are likely to be semi-structured in nature to allow for developing and emerging issues to be probed. Audio files of interviews will be securely held in password-controlled databases on Cardiff University servers. Digitally recorded interviews will be transcribed verbatim, anonymised using university-approved transcription procedures and checked by the researcher. No identifiable data will be published.

### Process evaluation analysis

Interview data will be subject to thematic analysis. Thematic analysis is an interpretive process, in which data are systematically searched for patterns to provide an illuminating description of the phenomenon [23]. This will involve the following stages: familiarisation with data, generating initial codes, searching, reviewing and defining themes [24]. Areas of contrast in participant perspectives, as well as similarity, will be identified. Qualitative coding software (such as NVivo10) will be used to assist in data analysis. Measures will be put into place to ensure validity and reliability. More than one researcher will be involved in the development of the coding framework and identification of themes (at global, organising and basic levels) [25]. Double coding will be carried out on a sample of the data until consensus is reached. Quantitative data on training session attendance gathered by facilitators and control group participant reports of usual care in follow-up assessment will inform sampling for qualitative interviews and be used as the basis for triangulation of collected data. An analysis plan for the process evaluation will be finalised prior to inception of the data collection.

### Public involvement

A contact group of carers in South Wales who received the intervention, but who are not participants in the trial, will provide lay input to the study. This will include guidance on participant materials and on-going carer engagement (e.g., retention). A face-to-face focus group discussion will take place with this contact group at least twice, supplemented by ad hoc contact via email at key trial milestones. The trial steering committee will include an independent lay representative with experience of foster care (i.e., being a carer).

### Other associated studies

Other associated planned studies include a process evaluation of implementing children’s skills groups in Wales and a process evaluation to explore the implementation of Fostering Changes training for residential care workers in Wales. There will also be a Feasibility Study of Fostering Healthy Futures, a programme developed in Colorado providing direct work-skills groups and mentoring to young people in foster care with the aim of improving longer-term mental health outcomes. All three studies will be led by the Children’s Social Care Research and Development Centre, Cardiff University, Wales, and conducted within the Confidence in Care project but outside of the trial.

## Discussion

The aim of the trial is to determine whether the Fostering Changes intervention can deliver important, significant differences to the way foster carers build positive relationships with their foster children, encourage positive child behaviour and set appropriate limits, compared with usual care. While positive benefit of the Fostering Changes programme has been found in the short term, this evaluation will produce robust evidence about the longer-term effectiveness of the Fostering Changes intervention for foster and kin carers and the process of implementing the programme. Beyond induction standards, there have been few attempts to provide evidence of specific interventions to support foster carers in their role. Fostering Changes is one programme with at least some short-term evidence; this study attempts to examine longer-term impact and in a broader, pragmatic setting. A scoping review examining potential interventions found that the Fostering Changes programme would be a good candidate for further evaluation [26]; the evaluation will provide the first evidence about the effectiveness of the Fostering Changes programme beyond the end of the training programme. The evaluation will contribute to the international evidence base on improving outcomes for looked-after children.

The evaluation will be run in social care settings. As noted in our introduction, trials are relatively rare in this context compared with healthcare settings [27] and running social care trials may provide particular challenges [28, 29]. The trial is also being conducted as part of a wider implementation of the Fostering Changes programme, where the Confidence in Care consortium are additionally charged with delivery to a large number of foster carers across Wales (at both trial and non-trial sites). Running a trial will require a large number of stakeholders to engage with the research, including intervention delivery staff, local authority social care and independent fostering provider staff, and professional research recruitment staff. It is unlikely that all stakeholders will be in equipoise about the trial intervention. That places a particular requirement on clear and balanced study information (and, where necessary, training) to ensure good adherence to the study protocol. In settings where provision of high-quality support for carers is not readily available, deferring the intervention for 12 months through random allocation may be viewed ethically contentious for families in need. Social workers are also time poor and asking them to assist in the recruitment to a training programme in addition to their daily workload may be challenging. Our recruiting (Health and Care Research Wales) research staff underwent additional training in the social care and foster care context; this training was developed and delivered in conjunction with the Confidence in Care delivery staff and sought to enable recruiters to better understand the social context and experiences of foster carers and increase their ability to engage with them at recruitment.

The study’s process evaluation includes an assessment of intervention implementation. This will bring together data collected as part of routine training delivery (such as sessional attendance records, training group participant satisfaction questionnaires), as well as data collected via research interviews. We will also explore the possibility of collecting direct observational data (either in-person or video-recorded) and developing a measure of intervention fidelity. While broad parameters for the process evaluation have been set, some novelties of this particular evaluation context mean that it will need to remain responsive to emerging implementation issues. We expect that some broader messages about trialling interventions in foster care settings may be a valuable secondary benefit of this trial.

## Additional file

Additional file 1: SPIRIT checklist. (DOCX 23 kb)

## Abbreviations

ANCOVA: analysis of covariance
CEQ: Carer Efficacy Questionnaire
CONSORT: Consolidated Standards of Reporting Trials
SDQ: Strengths and Difficulties Questionnaire
SOCSI: Cardiff University School of Social Sciences

## References

[1] StatsWales. Children looked after at 31 March by local authority and placement type. 2016. https://statswales.gov.wales/Catalogue/Health-and-Social-Care/Social-Services/Childrens-Services/Children-Looked-After/childrenlookedafterat31march-by-localauthority-placementtype. Accessed 7 Feb 2017.

[2] Meltzer H (2003). The mental health of young people looked after by local authorities in England.

[3] Jackson S (2010). Reconnecting care and education: from the Children Act 1989 to Care Matters. J Children’s Serv.

[4] Stein M (2012). Young people leaving care: supporting pathways to adulthood.

[5] Farmer E, Lipscombe J, Moyers S (2005). Foster care strain and its impact on parenting and placement outcomes for adolescents. Br J Soc Work.

[6] Holland S, Faulkner A, Perez-del-Aguila R (2005). Promoting stability and continuity of care for looked after children: a survey and critical review. Child Fam Soc Work.

[7] Biehal N (2010). Belonging and permanence: outcomes in long term foster care.

[8] National Assembly for Wales (2003). National Minimum Standards for Fostering Services.

[9] Turner W, Macdonald GM, Dennis JA (2007). Cognitive-behavioural training interventions for assisting foster carers in the management of difficult behaviour. Cochrane Database Syst Rev.

[10] Minnis H (2001). Mental health and foster carer training. Arch Dis Child.

[11] Macdonald G, Turner W (2005). An experiment in helping foster-carers manage challenging behaviour. Br J Soc Work.

[12] Briskman J (2012). Randomised controlled trial of the fostering changes programme, Research report DFE-RR237.

[13] Bandura A (1977). Social learning theory.

[14] Bowlby J (1998). A secure base: parent–child attachment and healthy human development.

[15] Pallett C (2002). Fostering changes: a cognitive-behavioural approach to help foster carers manage children. Adopt Foster.

[16] Scott S (2001). Multicentre controlled trial of parenting groups for childhood antisocial behaviour in clinical practice. BMJ.

[17] Goodman R (1999). The extended version of the Strengths and Difficulties Questionnaire as a guide to child psychiatric caseness and consequent burden. J Child Psychol Psychiatry.

[18] Haight WL (2005). Enhancing parent–child interaction during foster care visits: experimental assessment of an intervention. Child Welfare.

[19] Huitema BE (1980). Analysis of covariance and alternatives.

[20] Hamilton BL (1977). An empirical investigation of the effects of heterogeneous regression slopes in analysis of covariance. Educ Psychol Meas.

[21] Molenberghs G (2007). Editorial: what to do with missing data?. J R Stat Soc Series A-Stat Soc.

[22] Keith RE (2010). Fidelity of implementation: development and testing of a measure. Implemen Sci.

[23] Tesch R (1990). Qualitative research: analysis types and software tools.

[24] Braun V, Clarke V (2006). Using thematic analysis in psychology. Qual Res Psychol.

[25] Attride-Stirling J (2001). Thematic networks: an analytic tool for qualitative research. Qual Res.

[26] Holland S et al. Scoping study: transforming the outcomes for looked after children in Wales. 2013. https://cronfa.swan.ac.uk/Record/cronfa21009. Accessed 1 Mar 2017.

[27] Macdonald G (2008). Social work in the UK: a testing ground for trialists. J Child Serv.

[28] Mezey G (2015). Challenges to undertaking randomised trials with looked after children in social care settings. Trials.

[29] Dixon J (2013). Trials and tribulations: challenges and prospects for randomised controlled trials of social work with children. Br J Soc Work.

[30] Fostering Changes Training Centre. Fostering Changes programme. 2017 http://m.b5z.net/i/u/10067252/f/Publicity%20Materials/Fostering_Changes_Course_Overview_V1.pdf. Accessed 03 Mar 2017.

